# Effects of Corrugated Flat Rolling Process on the Bonding Interface, Microstructure, and Properties of Mg/Al Clad Plates

**DOI:** 10.3390/ma19020252

**Published:** 2026-01-08

**Authors:** Lifang Pan, Zhiyuan Zhu, Huanhuan Wang, Yong Chen, Sha Li, Cuirong Liu, Guangming Liu

**Affiliations:** 1 School of Materials Science and Engineering, Taiyuan University of Science and Technology, Taiyuan 030024, China; 2013017@tyust.edu.cn (L.P.); 18835389264@163.com (Z.Z.); wanghuanhuan@tyust.edu.cn (H.W.); 2022098@tyust.edu.cn (Y.C.); 2022107@tyust.edu.cn (S.L.); lcr@tyust.edu.cn (C.L.); 2 School of Engineering for Safety and Emergency Management, Taiyuan University of Science and Technology, Taiyuan 030024, China

**Keywords:** Mg/Al clad plate, composite rolling, finite element simulation, microstructure evolution, mechanical properties

## Abstract

In this paper, an AZ31B Mg/Al clad plate with 5052 aluminum alloy as the cladding was successfully prepared by a new composite process of corrugated roll roughing + flat roll finishing. First, finite element simulation software was used to predict and analyze the rolling process. Subsequently, experimental research was carried out according to the simulation results, and clad plate samples under single corrugated rolling and corrugated–flat rolling processes were prepared. Finally, the differences between the two clad plates in shape quality, interface bonding state, and mechanical properties were systematically compared and analyzed. The results show that, compared with the traditional corrugated rolling process, the sheet formed by corrugated–flat rolling composite rolling has a flatter shape with no warpage, and its interface bonding quality is better. The specific performance is as follows: the mechanical properties were significantly improved, and the tensile strength and elongation reached 259.96 MPa and 8.11%, respectively, in the transverse direction (TD). This study provides a new strategy for the preparation of high-performance Mg/Al clad plates.

## 1. Introduction

With advances in science and technology and the rapid development of the industrial sector, new technologies and new industries are constantly emerging, which puts forward newer, higher, and stricter requirements for the manufacturing industry. The manufacturing industry gradually regards energy conservation and emission reduction, green environmental protection, and resource conservation as development standards of enterprises, and related products of enterprises have gradually begun to develop towards lightweight and high-performance characteristics [1]. Therefore, the performance of a single traditional material has been unable to meet the needs of practical applications. In order to address these needs, material scientists have focused on the development of composite materials. As a new class of materials, composite materials are the key material basis for determining social progress. Double-layer and three-layer metal composites have become popular research directions in recent years, and lightweight design has become an important development direction for these composite materials [2].

Magnesium is a nonferrous light metal with many advantages, and the reserves of magnesium in the Earth’s crust are quite large, accounting for about 2.5% of the total amount of the Earth’s crust [3], indicating broad development and application prospects. Aluminum belongs to the nonferrous metal [4] series. At the same time, aluminum alloys are second only to steel in terms of usage among metal materials [5] due to their excellent comprehensive properties [6]. Based on the excellent properties of magnesium and aluminum, they can be made into composite materials [7], which are more suitable for industrial production and daily applications.

At present, there are various methods for preparing metal clad plates [8]. According to process attributes, these methods can be divided into the explosive welding composite method, the hot-pressing diffusion method, and the rolling composite method. Among them, the rolling composite method [9] has become the most widely used preparation method due to its high efficiency and wide applicability. In this method, a metal plate is deformed by applying pressure on the rolls, resulting in the rupture of the oxide film and work-hardened layer at the interface and exposing fresh metal matrices. Then, under continuous rolling force, the fresh metal is squeezed out from the cracks, which promotes the mutual diffusion of metal atoms on both sides and realizes the solid bonding of the interface.

Nie et al. [10] successfully prepared Al/Mg clad plates by the hot rolling composite method. Its tensile strength reached 230 MPa, far exceeding that of single-pass rolling plates (102 MPa), and the elongation reached 17%, showing excellent comprehensive mechanical properties. Wang et al. [11]. developed a rolling process combining corrugated rolls and flat rolls and systematically studied the evolution of the interface, microstructure, and properties of clad plates during two passes of rolling. The clad plate prepared by this process had good interface bonding quality and no defects. The tensile strength was 278.3 MPa, and the elongation was 16.91%. The performance was better than that of a single metal plate, and the shape was flat with no warpage. Li et al. [12] also prepared Al/Mg clad plates at 400 °C by using the combination technology of a corrugated roller and a flat roller. It was found that the interface was tightly bonded without voids, gaps, or intermetallic compounds, which further verified the improvement effect of the process on the interface quality and mechanical properties.

In summary, the rolling process plays a decisive role in the interface morphology, microstructure, and mechanical properties of Mg/Al clad plates. In this study, a new composite rolling strategy, combining rough rolling with corrugated rolls and finishing rolling with flat rolls, was proposed. Using this new method, a high-quality composite plate composed of a 5052 aluminum alloy layer and an AZ31B magnesium alloy substrate was successfully prepared. The resulting composites exhibit an excellent combination of lightweight properties, low density, high specific strength, and excellent interfacial integrity. The developed Mg/Al clad plate will have great application potential in the fields of aerospace and lightweight automobiles due to its excellent performance. It will also provide theoretical support and technical support for the further development and application of high-performance composite plate rolling.

## 2. Experimental Methods

### 2.1. Experimental Materials

The plates used in this study are commercial hot-rolled AZ31B magnesium and 5052 aluminum plates. The specific components of the experimental hot-rolled plates are shown in Table 1. The size of the magnesium alloy plate is 110 × 80 × 3 (mm), and the size of the aluminum alloy plate is 110 × 80 × 5 (mm).

### 2.2. Experimental Method

#### Finite Element Simulation

The modeling function of Deform3D software (Deform v13.0) cannot accurately draw the corrugated roller shape. At the same time, the assembly positioning in Deform3D is complicated, and the positioning is not accurate. Therefore, a three-dimensional model of the roll and rolling plate was established by Proe three-dimensional software, and the assembly function was used to assemble and locate the roll and rolling plate in Proe three-dimensional software. The model was then imported into Deform3D in STL format. Because the assembly positioning was carried out in Proe software, it was not necessary to re-locate in Deform, and the simulation value could be set directly.

**Figure 1:**
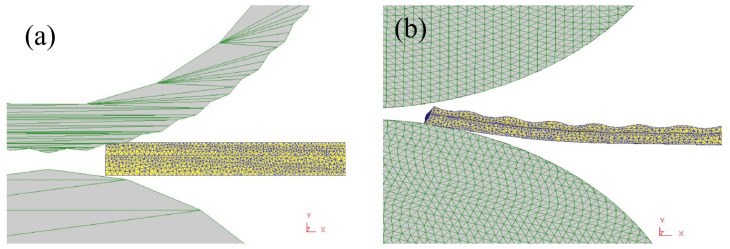
Establishment of the model: (**a**) corrugated roller; (**b**) flat rollers.

In Proe software, the nominal diameters of the corrugated roll and the flat roll in the first pass of corrugated rolling were both 150 mm, where the corrugated amplitude of the corrugated roll was 0.5 mm, the wave number was 100, and the length of the corrugated roll was 120 mm. In Deform software, the roll was meshed, and the minimum value was 1 mm. The sizes of the AZ31B and 5052 plates were 110 × 80 × 3 (mm) and 110 × 80 × 5 (mm), respectively. The plates were meshed in Deform3D, and the value was 0.4 mm. Material properties ALUMINUM-5052 [500–850 F (240 °C–440 °C)] with the material library in Deform3D software were selected. At the same time, according to the actual rolling situation, the boundary conditions of the roll and push plate were set. The rotation center coordinates and angular velocity were defined for the roll. The linear velocity direction of the roll rotation was the same as the motion direction of the rolling plate, and the value was the same. The relationship between the rolling plate and the roll was defined as a subordinate relationship. For the contact between the flexible body and the rigid body, the relevant friction coefficient was set. The shear friction between the roll and the rolled plate was set to be 0.3, the friction coefficient between the aluminum plate and the magnesium plate was set to be 0.5, the magnesium plate and the aluminum plate were set to be completely bound, and the interface heat transfer coefficient between the roll and the roll was 11 N/S/°C. The model is shown in Figure 1.

### 2.3. Rolling Experiment

The surfaces of the Mg plate and Al plate were first degreased using acetone. The contact interfaces of the Mg plate and Al plate were then ground with an angle grinder to expose fresh metal and remove any residual debris. Finally, the treated plates were securely bound at both ends with lead wires to prevent displacement or slippage during subsequent processing.

The resistance furnace was used to heat the billet before rolling. The heating temperature of the corrugated roll rolling in this experiment was 400 °C, and the heating time was 30 min. After the first-pass rolling, the rolled plate was quickly put into the resistance furnace and then heated at 400 °C for 15 min to prepare for the second-pass flat roll rolling. The rolling process parameters selected in this paper were as follows: For the first-pass corrugated roll rolling, the rolling temperature was 400 °C, and the rolling speed was 1.98 rad/s. For the second-pass flat rolling, the rolling temperature was 400 °C, the rolling speed was 1.98 rad/s, and the reduction was 35%.

### 2.4. Microstructure and Mechanical Property Experiment

The microstructure characterization and mechanical properties of the rolled plate were tested. After mechanical grinding and polishing, the metallographic samples were etched with the following solution: 1 mL nitric acid + 1 mL glacial acetic acid + 1 g oxalic acid + 150 mL distilled water. The method was wipe, and the etching time was 10 s. At the same time, the microstructure and tensile fracture of the Mg/Al interface were observed by a scanning electron microscope (SEM, Zeiss Sigma 300, Jena, Germany). The new phases produced at different positions of the Mg/Al interface were determined by an Oxford X-ray energy dispersive spectrometer (EDS, Zeiss Sigma 300, Germany) probe. After polishing, the samples were electrolyzed with 10% perchloric acid solution at a voltage of 45 V for 1 min, and the samples were analyzed by electron backscatter diffraction (EBSD, Zeiss Sigma 300, Germany). The mechanical properties of the Mg/Al clad plate at room temperature were tested by a microcomputer-controlled electronic universal testing machine (DNS200, made by Kimlida Electronic Technology Co., Ltd., Guangzhou, China). The sampling directions were rolling direction (RD) and transverse direction (TD), and the stretching speed was 0.5 mm/min. Each data point was subjected to three tensile tests, and the average value was taken to ensure the accuracy of the experimental results.

## 3. Results and Discussion

### 3.1. Simulation Results Analysis

**Figure 2:**
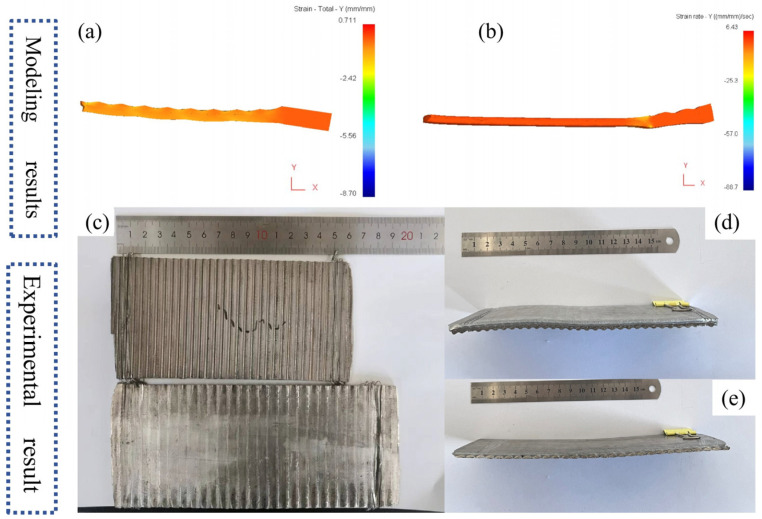
Plate type comparison: (**a**) corrugated rolling; (**b**) flat rolling. (**c**–**e**) Results of rolling experiments.

As shown in Figure 2, the shape of the clad plate after corrugated–flat roll finishing is significantly improved, and its warpage is much lower than that of the clad plate rolled by a single corrugated roll. The physical plate type prepared based on the simulation parameters is consistent with the predicted results, indicating that the composite rolling process effectively eliminates warpage and obtains a flat clad plate.

**Figure 3:**
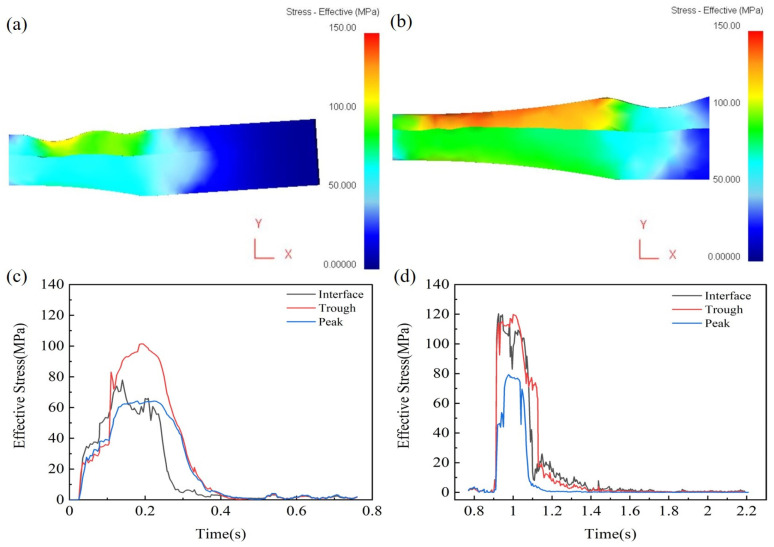
Effective strain of Mg/Al clad plates: (**a**,**c**) corrugated clad plate; (**b**,**d**) corrugated–flat rolled clad plate.

In the process of hot rolling the Mg/Al clad plate, the stress state between the clad plate and the roll and between the magnesium and aluminum double layers has an important influence on the forming quality of the clad plate. It is of great significance to analyze the stress distribution in the rolling process. Figure 3 shows that the equivalent stress in the corrugated-rolled clad plate is higher at the trough (approximately 115 MPa) than at the peak (around 90 MPa). Figure 3b indicates that during corrugated–flat rolling, the highest equivalent stress occurs in the contact region between the magnesium layer and the roll. The elevated stress levels facilitate the fragmentation of the work-hardened layer and oxide film at the interface, promoting the exposure of fresh metal and thereby improving interfacial bonding [13].

**Figure 4:**
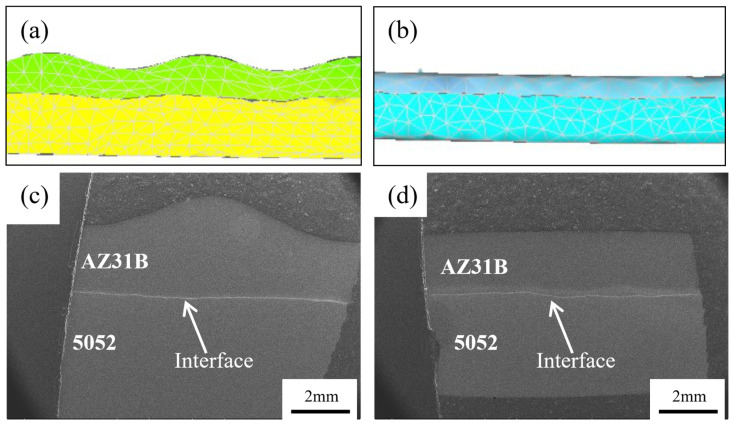
Interface morphology of Mg/Al clad plates. (**a**) Simulation results of a corrugated composite plate. (**b**) Simulation results of a corrugated–flat-rolled clad plate. (**c**) Experimental results of a corrugated composite plate. (**d**) Experimental results of a corrugated–flat-rolled clad plate.

Figure 4a,b show that the interface of the corrugated rolling and corrugated–flat rolling clad plate is slightly corrugated. This morphology changes the bonding surface from a traditional two-dimensional plane to a three-dimensional space structure, increasing the metal contact area and thereby effectively improving the interface bonding quality [14].

### 3.2. Interface Morphology and Element Diffusion Analysis

**Figure 5:**
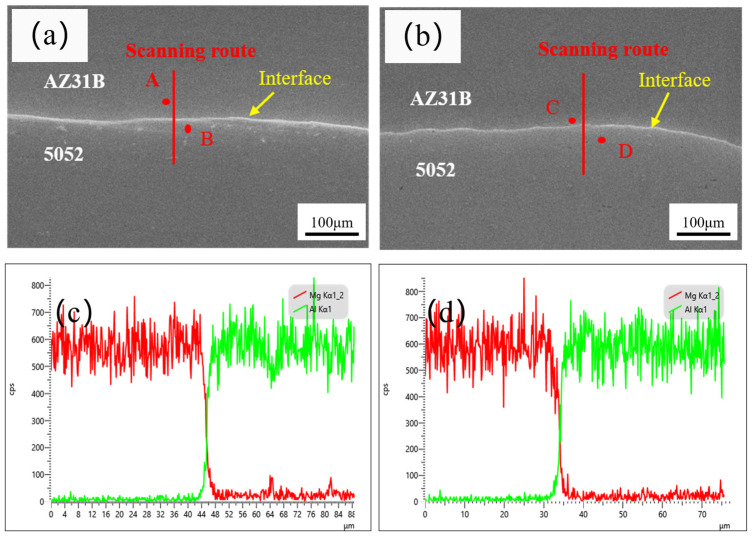
SEM at the interface of a Mg/Al clad plate: (**a**,**c**) corrugated rolling; (**b**,**d**) corrugated–flat rolling.

Figure 5 presents the SEM morphology and corresponding EDS line scanning profile across the interface of Mg/Al clad plates fabricated by corrugated rolling and corrugated–flat rolling processes. As shown in Figure 5a,b, a sound bonding interface with a wavy morphology is achieved between the Mg/Al plates, free from defects such as voids, cracks, or delamination. The formation of this wavy interface can be attributed to the differential flow behavior between Mg and Al during rolling, combined with the fragmentation of the pre-rolling grinding-induced hardened layer on the corrugated surface under rolling pressure. This process facilitates the interpenetration of freshly exposed metal atoms into surface microcracks, enhancing interfacial bonding.

Figure 5 is the EDS point scanning analysis near the interface of corrugated rolling and corrugated–flat rolling. Figure 5a and Figure 5b are the results of the magnesium side and aluminum side of the corrugated rolling interface, respectively. Mg at point A is 87.06 (in at. %), Al at point B is 12.94 (in at. %), Mg at point C is 94.96 (in at. %), and Al at point D is 5.04 (in at. %). The results of point scanning show that there is indeed mutual diffusion between elements near the interface during corrugated rolling and corrugated–flat rolling.

Figure 5c,d display the EDS line scanning results at the corresponding interfaces, with the red and green curves representing the distribution of Mg and Al elements, respectively. The analysis reveals a sharp decline in Mg concentration from the magnesium side toward the aluminum side, while the Al content exhibits an inverse distribution pattern. The measured thickness of the interfacial diffusion layer is approximately 6.2 μm for the corrugated-rolled sample and about 8.33 μm for the corrugated–flat-rolled sample, confirming the occurrence of mutual diffusion between Mg and Al and the establishment of a metallurgical bond at the interface. Based on the gradual concentration transitions observed in the EDS profiles, no evidence suggests the formation of intermetallic compounds (IMCs) at the interface in either processing route.

**Figure 6:**
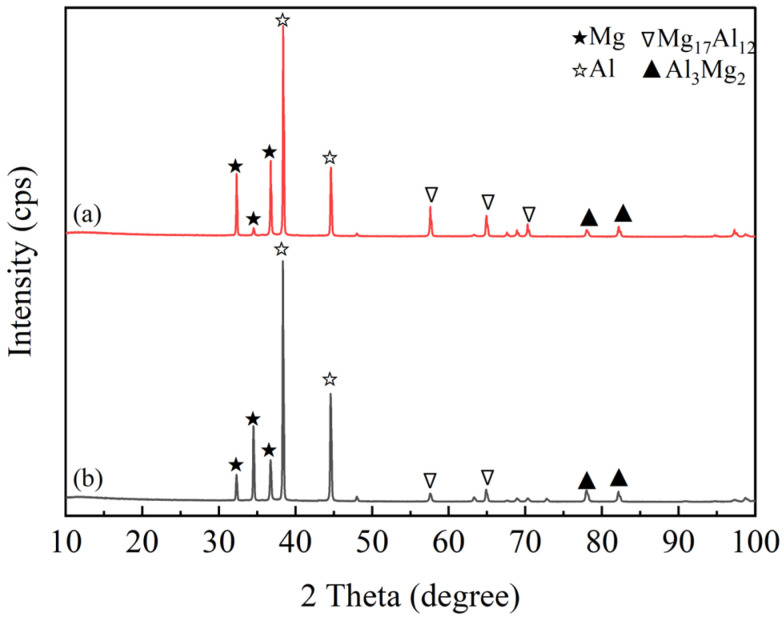
The XRD patterns of a composite sheet: (**a**) corrugated rolling; (**b**) corrugated–flat rolling.

Figure 6 is the XRD experiment near the interface of the corrugated rolling and corrugated–flat rolling clad plate, and the phase composition at the interface after two passes of rolling is analyzed and determined. Observing Figure 6, it is found that after corrugated roll rolling and corrugated–flat rolling, in addition to the Mg phase and Al phase, Al_3_Mg_2_ and Mg_17_Al_12_ phases are found at the interface of the Mg/Al clad plate. The main reason for this phenomenon is that in the process of severe plastic deformation, a new surface, high-density defects, and a local temperature rise will be generated at the interface, which provides a strong thermodynamic driving force and kinetic conditions for atomic interdiffusion and interface reactions. At the same time, in the process of high-pressure and short-time deformation, a steep composition gradient from Al-rich to Mg-rich is formed on both sides of the interface. The Al_3_Mg_2_ phase tends to nucleate on the Al-rich side, while the Mg_17_Al_l2_ phase tends to nucleate on the Mg-rich side.

### 3.3. Microstructure Analysis of As-Rolled Mg/Al Clad Plate 

#### Metallographic Analysis of Mg/Al Clad Plate in Rolling State

**Figure 7:**
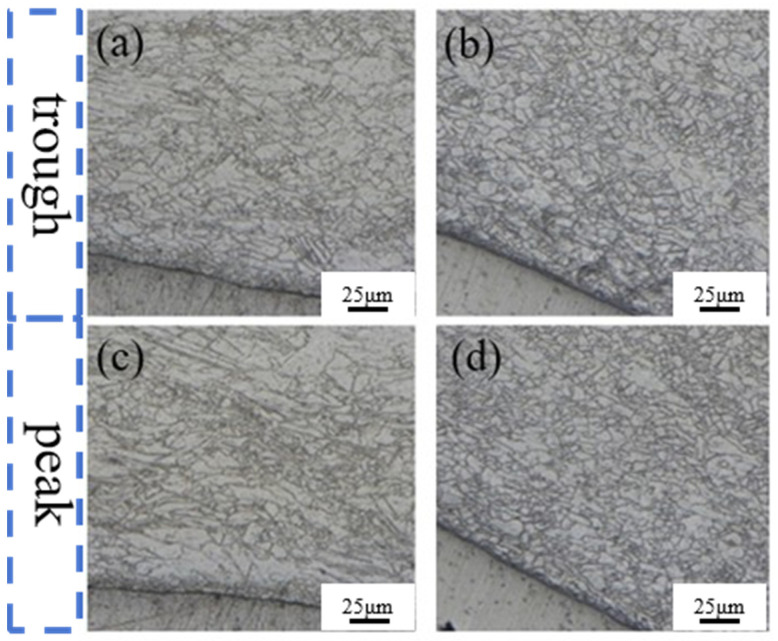
Metallographic diagram of an AZ31B component plate in a Mg/Al clad plate: (**a**,**c**) corrugated trough and crest; (**b**,**d**) corrugated–flat rolling trough and crest.

Figure 7 displays the metallographic structure on the Mg side of the interface in both the corrugated-rolled and corrugated–flat-rolled clad plates. As observed in Figure 7a, the original coarse grains are elongated along the rolling direction, exhibiting a chain-like distribution. A distinct shear band, oriented at approximately 45° to the rolling direction, has formed, in which the grains are noticeably refined. This microstructural evolution results from the heterogeneous plastic deformation experienced by the magnesium plate during corrugated rolling.

According to A. Galiyev’s [15] research, such necklace-like fine grains are formed by subgrain nucleation and growth. At the same time, there are a large number of cross-distributed twins in the microstructure [16], and fine grains are distributed along the twin boundary, indicating that twinning-induced recrystallization occurs. This phenomenon is related to the too-fast cooling rate during the rolling process, which leads to the failure of dynamic recrystallization grains to grow sufficiently [17].

Figure 7a,b show that the interface region is dominated by a fine-grained structure. This is because Al has a face-centered cubic structure and 12 actuated slip systems at room temperature, showing excellent plastic coordination ability and uniform deformation ability. During the rolling process, high-density dislocations easily accumulate and rearrange, providing sufficient driving force and nucleation points for subsequent dynamic recrystallization. In contrast, Mg has a close-packed hexagonal structure, and the slip system that can be started at room temperature is limited (mainly basal slip). The plastic deformation is more dependent on twinning, and its dislocation storage efficiency and uniformity are low, which limits the nucleation rate and refinement effect of dynamic recrystallization. Therefore, there is more significant grain refinement on the Al side. When magnesium is rolled at 400 °C, both basal slip and non-basal slip are activated. Although the number of slip systems increases, its plastic flow stress is still lower than that of aluminum. Under the coupling effect of rolling force and torque, the interface area bears a large shear stress, resulting in increased friction and temperature, thus promoting the refinement of the original grains on the magnesium side [18].

There are many long strip deformation grains in Figure 7a, and the number of twins is less than that in Figure 7b, which is related to the fact that the deformation at the trough caused by the special roll shape of the corrugated roller is greater than that at the peak. After the second pass of flat rolling, Figure 7b,c show that a large number of twins and shear bands disappear, and twins are ‘devoured’ by fine grains. The microstructure is dominated by fine recrystallized grains, while larger grains exist in the form of twins. This is attributed to the preheating treatment of 400 °C × 15 min immediately after corrugated rolling, which promotes the growth of recrystallized grains to a certain extent. The subsequent flat rolling process triggers dynamic recrystallization again, further refines grains, and improves microstructure uniformity.

### 3.4. Microstructure Analysis of the Mg/Al Clad Plate Interface

**Figure 8:**
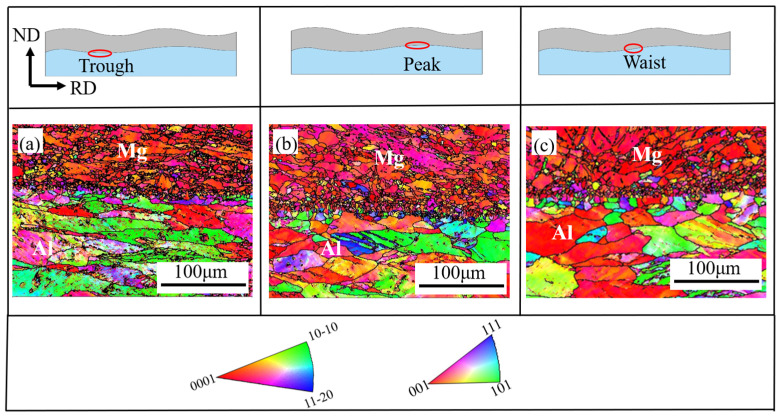
IPF maps of a corrugated rolling sheet at different positions: (**a**) trough; (**b**) crest; (**c**) waist.

Figure 8a–c present the inverse pole figure (IPF) maps of the Mg/Al clad plate interface processed by corrugated rolling, corresponding to the trough, peak, and waist regions, respectively. The results demonstrate tight bonding at all three interfacial locations, with no observable defects, such as gaps, voids, or reaction diffusion layers, confirming the high quality of the interfacial bonding.

The rolling process is an asymmetric [19] deformation, resulting in an uneven distribution of shear strain at the interface of the clad plate: the shear strain is the largest at the trough and the smallest at the peak. This strain gradient helps to change the interface stress state and promote local plastic strain [20]. Under the combined action of compression and shear strain, the friction in the interface deformation zone is intensified, the temperature rise is significant, and more slip systems are activated, thereby promoting the occurrence of dynamic recrystallization in the Mg and Al interface region [21] and forming a fine-grained structure.

### 3.5. Analysis of Mechanical Properties of Mg/Al Clad Plate in Rolling State

#### Mechanical Tensile of Mg/Al Clad Plate

**Figure 9:**
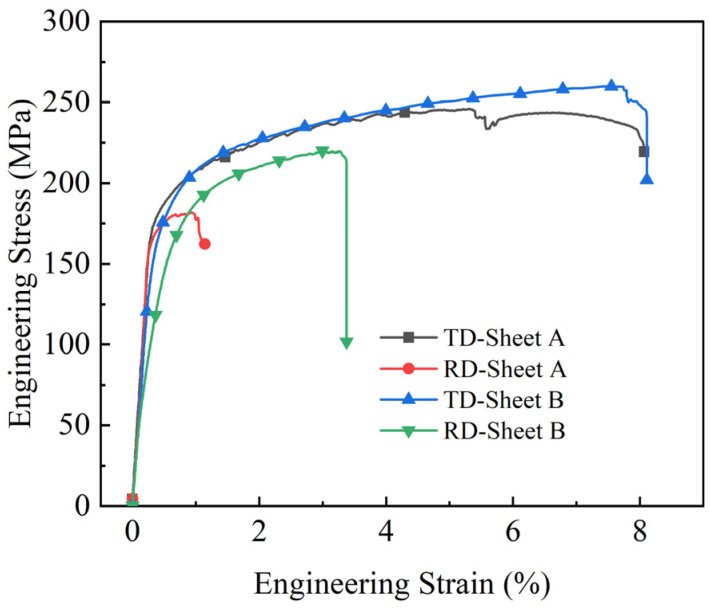
Engineering stress–strain curves of Mg/Al clad plates.

Figure 9 displays the tensile curves of Mg/Al clad plates fabricated by corrugated rolling (Sheet A) and corrugated–flat rolling (Sheet B), tested along the transverse (TD) and rolling (RD) directions. As shown, both clad plates exhibit significant mechanical anisotropy, with superior tensile properties observed in the TD direction compared to the RD direction.

Specifically, the corrugated-rolled clad plate exhibits the poorest comprehensive mechanical properties along the RD direction, with an ultimate tensile strength of 181.76 MPa and an elongation of only 1.76%, demonstrating nearly no plastic deformation prior to fracture. In contrast, the TD direction shows significantly improved properties, with an ultimate tensile strength of 245.83 MPa and an elongation of 7.91%. The corrugated–flat-rolled clad plate also displays similar anisotropic behavior, with superior comprehensive mechanical properties in the TD direction compared to the RD direction. Specifically, the TD direction exhibits a tensile strength of 259.96 MPa and an elongation of 8.11%, whereas the RD direction shows corresponding values of 220.19 MPa and 3.37%, respectively. Notably, the elongation in the TD direction is 140% higher than that in the RD direction.

The main reason for the anisotropy phenomenon, where the elongation in the TD direction is significantly higher than that in the RD direction, is as follows: Microstructural analysis reveals that the magnesium alloy in this direction contains a high density of fine grains and twins. Twinning, which results from hindered dislocation motion, is typically activated under stress concentration when slip systems are inadequate. The heterogeneous distribution of twin structures further promotes localized stress concentration, thereby deteriorating room-temperature mechanical performance.

In summary, the Mg/Al clad plate prepared by the two rolling processes has obvious mechanical anisotropy. The performance difference in the RD direction is mainly affected by grain size, degree of recrystallization, and twins, which is also common in single-metal rolled plates.

### 3.6. Analysis of Fracture Appearance

**Figure 10:**
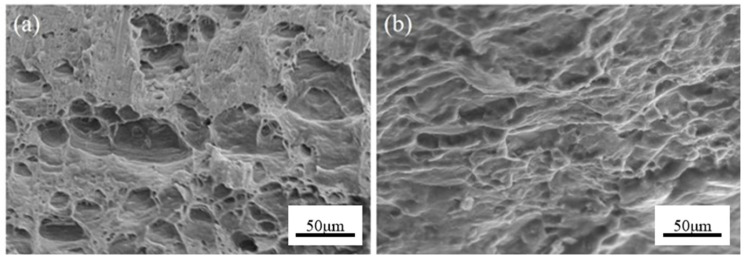
RD direction tensile fracture of a Mg/Al clad plate: (**a**) Al side; (**b**) Mg side.

Figure 10 is the tensile fracture morphology of the Mg/Al clad plate in the RD direction. It can be seen from Figure 10a that the number of dimples on the aluminum side fracture is small, the size is large, and there is an obvious smooth plane, which is characterized by brittle fracture. Figure 10b shows that the number of dimples on the magnesium side fracture is also small, the size is very small, the depth is shallow, and the boundary is blurred, reflecting its limited local plastic deformation ability.

This is because the tensile fracture of the material is essentially the result of dislocation motion along the slip system. Under the action of axial tensile stress, stress concentration easily occurs at defects, such as precipitated phases, grain boundaries, and dislocation aggregation in the material, and then, microcracks or micropores are formed. For the Mg/Al clad plate prepared in this study, the fracture behavior is significantly controlled by the Mg/Al interface characteristics. In the hot rolling process, the hard, brittle intermetallic compound layer formed at the interface becomes the weakest link. Under tensile stress, due to the huge plastic mismatch between the IMC layer and the magnesium and aluminum substrates on both sides, dislocations are seriously piled up here, resulting in the interface IMC layer or its three-phase point becoming the core initiation area of microcracks. Subsequently, under the action of shear stress [22], these microcracks rapidly expand along the brittle interface and connect with the microvoids in the matrix, eventually leading to early fracture dominated by interfacial debonding, which significantly reduces the macroscopic plasticity of the material.

## 4. Conclusions

In this study, a systematic investigation was conducted on Mg/Al clad plates fabricated by corrugated rolling and corrugated–flat rolling in the as-rolled condition, leading to the following main conclusions:

(1) The finite element simulation results indicate that the clad plate produced by corrugated–flat rolling exhibits superior shape quality without warpage, compared to that fabricated by conventional corrugated rolling. Based on the simulation parameters, actual clad plates were successfully manufactured, showing high consistency in plate shape with the simulated results. This agreement validates the practical utility of the finite element model in guiding the corrugated–flat rolling process.
(2) The interface analysis shows that the corrugated–flat rolling process can achieve excellent metallurgical bonding, and there are no pores, cracks, or delamination defects at the interface. Both sides of magnesium and aluminum in the interface area show grain refinement characteristics, and the grain size distribution is position-dependent: the grain size in the trough is the smallest, the peak is the largest, and the waist is between the two. This distribution is directly related to the non-uniform strain field during the corrugated rolling process. The shear strain is the largest at the groove and the smallest at the peak, resulting in a continuous gradient change in the degree of recrystallization along the interface.
(3) The prepared composite plate exhibits significant mechanical anisotropy, and the lateral performance is better than the rolling direction. The tensile strength of the transverse specimen is 259.96 MPa, and the elongation is 8.11%. The samples in the rolling direction are 220.19 MPa and 3.37%, respectively, and the transverse elongation is about 140% higher than that in the rolling direction. This anisotropy can be attributed to the combined effect of the interface ripple geometry on the load transfer path and the grain morphology texture. The transverse specimen has a more uniform stress distribution and stronger necking resistance during the tensile process.

## Figures and Tables

**Table 1:** Chemical constituents of the experimental hot-rolled plates (wt.%).

	Mg	Cr	Ni	Fe	Mn	Si	Cu	Zn	Al
AZ31B	rest	-	0.005	0.005	0.50	0.04	-	0.90	3.01
5052	2.2~2.8	0.15~0.35	-	0.40	0.10	0.25	0.10	0.10	rest

## Data Availability

The original contributions presented in this study are included in the article. Further inquiries can be directed to the corresponding author.
